# Validity and Reliability of Persian Version of HIV/AIDS Related Stigma Scale for People Living With HIV/AIDS in Iran 

**Published:** 2015-11

**Authors:** Davoud Pourmarzi, Ashraf Khoramirad, Hoda Ahmari Tehran, Zahra Abedini

**Affiliations:** 1 Department of Epidemiology, Reproductive Health Research Center, Guilan University of Medical Sciences, Rasht, Iran; 2 Department of Nursing, Faculty of Nursing and Midwifery, Qom University of Medical Sciences, Qom, Iran; 3 Department of Medical Education, M.sc in Midwifery, PhD Student of Medical Education, Tehran University of Medical Sciences, Tehran, Iran

**Keywords:** HIV/AIDS, Stigma, Validity, Iran

## Abstract

**Objective:** To assess the perceived HIV/AIDS related stigma a comprehensive and well developed stigma instrument is necessary. This study aimed to assess validity and reliability of the Persian version of HIV/AIDS related stigma scale which was developed by Kang et al for people living with HIV/AIDS in Iran.

**Materials and methods:** Thescale was forward translatedby two bilingual academic members then both translations were discussed by expert team. Back-translation was done by two other bilingual translators then we carried out discussion with both of them. To evaluate understandability the scale was administered to 10 Persons Living with HIV/AIDS (PLWHA). Final Persian version was administered to 80 PLWHA in Qom, Iran in 2014. Test–retest reliability was assessed in a sample of 20 PLWHA after a week by intra-class correlation coefficient (ICC).

**Results:** Cronbach’s alpha coefficient for overall scale was 0.85. Also Cronbach’s alpha coefficients for the five subscales were as follows: social rejection (9 items, α = 0.84), negative self-worth (4 items, α = 0.70), perceived interpersonal insecurity (2 items, α = 0.57), financial insecurity (3 items, α = 0.70), discretionary disclosure (2 items, α = 0.83). Test–retest reliability was also approved with ICC = 0.78. Correlation between items and their hypothesized subscale is greater than 0.5. Correlation between an item and its own subscale was significantly higher than its correlation with other subscales.

**Conclusion:** This study demonstrate that the Persian version of HIV/AIDS related stigma scale is valid and reliable to assess HIV/AIDS related stigma perceived by people living whit HIV/AIDS in Iran.

## Introduction

Still after more than two decades of the pandemic of HIV/AIDS the stigma attributed to this disease remains a significant challenge for HIV/AIDS care programs (1). People living with HIV/AIDS (PLWHA) often suffer from stigma and the emotional, mental, financial, and social burden of the disease (2-4). Stigma is a powerful social phenomenonand involves vulnerable populations. Stigma is defined as a mark of disgrace and shame associated with specific conditions (5). Perceived stigma can be assessed internally and externally. Negative self-worth, perceived interpersonal insecurity and discretionary disclosure reflects internal stigma and social exclusion and lack of financial security reflects external stigma (4, 6).

HIV/AIDS-related stigma is not only a violation of human rights, but also contributes to the prevention and control of HIV/AIDS programs by blocking their progress and success (3). Stigma in labeled people lead to discrimination, loss of social status and coping behaviors, reduced quality of life, exacerbations, discontinued treatment, and the ravages of the family(3, 4, 7).Previous studies have reported that high levels of HIV/AIDS-related stigma are strongly associated with high levels of depression and low self-efficacy (7-10).

HIV/AIDS-related stigma and discrimination is established by many factors, including lack of awareness of HIV/AIDS spatially transmission routs, lack of access to treatment, the incurability of AIDS, and prejudices related to certain actions associated with HIV such as sexual activities, substance abuse, addiction, illness and death (3, 4).

In Iran, the status of the HIV/AIDS epidemic has proceeded from low-prevalence into the concentrated phase, so during the past twenty years, the number of patients has grown significantly (11-13).In Iran according to the latest published statistics, about 29000 HIV/AIDS cases have been detected and it is estimated that these cases are only about half of Iranian HIV infected persons (12). High level of discrimination and stigma associated with HIV/AIDS has led to self-imposed restrictions in the treatment of Iranian HIV infected people (12, 14, 15). Iranian patients infected with HIV/AIDS mostly fear the stigma and ostracism from family that is associated with this disease. They also have a sense of insecurity about job loss, shelter and relationships and it seems they experience a lot of stress and psychological problems after diagnosis (15).

Although it is evident that this stigma is the major obstacle to an effective response to the HIV/AIDS epidemicand HIV/AIDS-related stigma elimination is targeted by international organizations, efforts to reach this goal are not effective. The complexity of HIV/AIDS-related stigma is often known as the main reason for the limited response to the phenomenon (1). The first step of planning any program for the elimination of HIV/AIDS-related stigma is identifying the burden of the stigma and its domains. To assess perceived HIV/AIDS-related stigma a well-developed stigma instrument is necessary (16). 

In Iran, a few studies have been conducted to validate the HIV/AIDS stigma scales. SyedAli Naghi et al., translated and used a stigma index to evaluate HIV/AIDS perceived stigma among patients living with HIV/AIDS in six cities in Iran. But that study was not a validity and reliability assessment study (17). Another study in Iran evaluated validity and reliability of the Iranian version of the 16-item HIV/AIDS Stigma Instrument–PLWHA (HASI-P) and assessed the internalized stigma in three domains including blaming and distancing, discrimination, and fear. The authors of that study concluded that the scale’s briefness can make the HIV/AIDS stigma appear as a single-dimensional scale, while this stigma is normally considered to be a multi-dimensional construct (18).

Fife and Wright developed a scale to measure the perceived external and internal stigma in persons living with HIV/AIDS and cancer (19). The scale was revised by Kang et al. for use among Asians and Pacific Islanders living with HIV/AIDS in the United States (6). 

In our study we used the revised scale that consists of 20 items and its good size measures internal and external HIV/AIDS stigma in five subscales: social rejection (9 items), negative self-worth (4 items), perceived interpersonal insecurity (2 items), financial insecurity (3 items), and discretionary disclosure (2 items). In this scale social rejection and financial insecurity subscales represented external stigma; negative self-worth, perceived interpersonal insecurity, and discretionary disclosure represented the internal stigma (6).

This study aimed to translate and validate the Persian version of Kang et al HIV/AIDS related stigma scale

 for people living with HIV/AIDS in Iran.

## Materials and methods

***Forward translation***

The translation and validity of the HIV/AIDS stigma scale was conducted in four stages. In the first stage two bilingual nursing lecturers of the Qom University of Medical Sciences translated the scale independently, with one acting as the principle researcher. Then the two versions and original English version were discussed in an expert team to create a Persian version. The expert team members included a lecturer of community nursing, a lecturer of psychiatric nursing, an epidemiology from the Qom University of Medical Sciences, and two mental health counselors and one general practitioner from the Qom Consulting Clinics of Behavioral Disorders. In the panel each item and its two proposed translations were read aloud and experts discussed them and the suggested responses. Based on the experts’ suggestions an initial Persian version was produced.

***Backward translation***

The initial Persian version was back-translated into English by two bilingual translators, who were faculty members of the Qom University of Medical Sciences and had not previously seen the original scale. The principle researcher compared the original version and back-translated version and then by discussions held among the principle researcher and two translators any discrepancy or variations between them were resolved. Some changes were made in the Persian version based on outcomes of those discussions.

***Understandability of the questionnaire***

The modified Persian version scale after the backward-translation process was administered to 10 persons living with HIV/AIDS (PLWHA) for evaluating understandability. The instrument was filled out by interviews by a coworker. The interviewer read each item to the participants and asked them about clarity and simplicity of the words and sentences.

***Psychometric testing***

The final Persian version was tested for reliability and validity. A cross-sectional study was conducted on 80 PLWHA who attended the Consulting Clinics of Behavioral Disorders in Qom, Iran from September 2014 to January 2015. Qom Consulting Clinics of Behavioral Disorders is the only clinic that provides services for all PLWHA in Qom and is affiliated with the Qom University of Medical Sciences. All provided services are free. PLWHA who were diagnosed at least one month ago and were at least 18 years old were eligible to participate in our study. Patients who suffered from any major psychological disorder were excluded. Sampling was performed using the convenience sampling method considering limited access to PLWHA. In order to find study participants a member of the research team approached potential patients who attended the Clinic to receive services during the study period and all potential subjects were invited to participation. After receiving informed consent forms from all the participants the subjects themselves filled out the questionnaires. For illiterate participants (two participants) one of our coworkers read the items and possible answers and filled out the questionnaires based on the participants' responses.

Psychometric testing was carried out on 80 completed HIV/AIDS-related stigma scale.

***Ethical consideration***

This study was approved by the ethical committee of the Qom University of Medical Sciences (NO: IR.MUQ.REC.1394.91). Participation in this study was voluntary and all questionnaires were anonymous.

***Reliability***

Scale reliability was tested by performing internal consistency analysis. Cronbach’s alpha coefficient was computed for the scale and its five subscales to measure internal consistency. Also test–retest reliability was assessed in a sample of 20 PLWHA after a week by intra-class correlation coefficient (ICC). ICCs ≤ 0. 4 was considered poor to fair, 0.41–0.60 moderate, 0.61–0.80 good, and > 0.80 excellent (Bartko, 1966).

***Validity***

Scale validity was tested by assessing convergent and discriminant validity. Pearson’s correlation coefficients were calculated for each item with its own subscale and with other subscales. Convergent validity was passed when correlation coefficients were greater than 0.4. Correlation between an item and its own subscale greater than the correlation between that item and another subscale was considered satisfactory in the test of discriminant validity .For statistical analysis SPSS software version 21 was used.

## Results

***Forward translation***

Eleven items in both forward translations were identical, although there were some differences in the wording of the other items.

***Expert panel***

Experts accepted 14 translated items without any changes. Other items were modified based on the panel’s suggestions. Also the team suggested including the neutral option in the response option. Then participants rated their extended agreement with each item by a 5-point Likert type scale. Points were assigned as follows: 1= strongly disagree, 2 = disagree, 3 = neutral, 4 = agree, 5= strongly agree. Total possible scores ranged from 20 to 100. The higher score showed a stronger sense of feeling stigmatized.

***Backward translation***

Sixteen items were back-translated almost identically by both translators. After comparing the back-translated items and original version some discrepancies were detected in 10 items. After discussion with the two back-translators, 12 items were approved without any changes and for the other items some revisions were done based on the translators’ comments.

***Understandability of the questionnaire***

None of the participants mentioned unclear or difficult words or sentences. Hence, all the items passed the understandability evaluation.

***Psychometric testing***

Data from 80 completed questionnaires were analyzed. The mean age of the participants was 33.01 ± 7.99 and most participants (57.7%) were male. The majority of participants were never married (50.0%), resided in cities (93.4%), and jobless (51.9%). All most half of the subjects had a high school diploma (46.3%). The prevalent rout of infection (46.3%) was sharing needles. Time post HIV diagnosis in most of the patients (62.5%) was less than one year (Table 1). 

Mean score of perceived stigma was 72.04 ± 12.08. Means score of perceived stigma in social rejection, negative self-worth, perceived interpersonal insecurity, financial insecurity, and discretionary disclosure subscales were 33.51 ± 6.95, 12.60 ± 3.87, 8.08 ± 1.81, 9.49 ± 2.89, and 8.36 ± 1.92, respectively. Means score of external and internal perceived stigma were 43.00 ± 8.53 and 29.04 ± 5.34, respectively.

***Reliability and Validity***

Cronbach’s alpha coefficient for the overall scale was 0.85. Also Cronbach’s alpha coefficients for the five subscales were as follows: social rejection (9 items, α = 0.84), negative self-worth (4 items, α = 0.70), perceived interpersonal insecurity (2 items, α = 0.57), financial insecurity (3 items, α = 0.70), and discretionary disclosure (2 items, α = 0.83). Test–retest reliability was also approved due to a good ICC values (ICC = 0.78).

**Table 1:** Characteristics of participants

**Variable **		**Summary statistics**
Mean age (SD)		33.01 ± 7.99 (n = 80)
Gender	Male	45 (57.7%)
	Female	33 (42.3%)
Marital status	Married	18 (26.5%)
	Single	34 (50%)
	Divorced	8 (10%)
	Widowed	8 (10%)
Job	Jobless	40 (51.9%)
	Worker	8 (10.4%)
	Self-employed	21 (27.3%)
	Housewife	8 (10.4)
Place of residence	Urban	71 (93.4%)
	Rural	5 (6.3%)
Education level	Illiterate or Primary	20 (25%)
	Secondary	17 (21.3%)
	High school	37 (46.3%)
	University	6 (7.5%)
Rout of infection	Sharing needles	37 (46.3%)
	Sexual contact	29 (36.3%)
	Receiving blood	4 (5%)
	Others	10 (5%)
Time post-HIV diagnosis (year)	˂ 1	50 (62.5%)
	1-4	29 (36.3%)
	≥ 4	1 (1.3%)

The correlation between items and their hypothesized subscale were higher than 0.5 for all items. The minimum coefficient was 0.53 for item 1 and the maximum was 0.93 for item 19. Correlation between an item and its own subscale was significantly higher than its correlation with other subscales. Also, correlation between an item and scale other than its hypothesized scale were lower than the correlation between that item and its hypothesized scale, so all items passed the test for convergent and discriminant validity (Table 2).

**Table 2:** Item-scale correlation of HIV/AIDS related stigma scale

HIV/AIDS related stigma scale	Items	Social rejection	Negative self-worth	Perceived interpersonal insecurity	Financial insecurity	Discretionary disclosure
**Social rejection**	**1. My employer/coworkers have discriminated against me because of my illness**	**0.533**	**0.321**	**0.119**	**0.304**	**-0.215**
	**2. Some people act as though I am less competent than usual**	**0.737**	**0.459**	**0.260**	**0.615**	**0.288**
	**3. I feel that I have been treated with less respect than usual by others**	**0.732**	**0.116**	**0.258**	**0.151**	**0.081**
	**4. I feel others are concerned they could ‘‘catch’’ my illness through contact like a handshake or eating food I make**	**0.732**	**0.105**	**0.2338**	**0.126**	**-0.041**
	**5. I feel others avoid me because of my illness**	**0.718**	**0.022**	**0.202**	**0.104**	**0.442**
	**6. Some family members have rejected me because of my illness**	**0.795**	**0.288**	**0.227**	**0.456**	**0.144**
	**7. I feel some friends have rejected me because of my illness**	**0.546**	**0.385**	**0.179**	**0.237**	**0.169**
	**8. I encounter embarrassing situations as a result of my illness**	**0.590**	**0.013**	**0.290**	**0.109**	**0.120**
	**9. Due to my illness, others seem to feel awkward and tense when they are around me**	**0.571**	**-0.109**	**0.315**	**0.099**	**0.153**
**Negative self-worth**	**10. I feel I am at least partially to blame for my illness**	**0.109**	**0.718**	**-0.097**	**0.251**	**-0.110**
	**11. I feel less competent than I did before my illness**	**0.277**	**0.837**	**0.129**	**0.392**	**0.037**
	**12. Due to my illness, I sometimes feel useless**	**0.138**	**0.732**	**0.031**	**0.487**	**0.301**
	**13. Changes in my appearance have affected my social relationships**	**0.304**	**0.624**	**0.282**	**0.391**	**0.286**
**Perceived interpersonal insecurity**	**14. I feel I need to keep my illness a secret**	**0.385**	**0.127**	**0.832**	**0.114**	**0.419**
	**15. I have a greater need than usual for reassurance that others care about me**	**0.204**	**0.075**	**0.843**	**0.283**	**0.109**
**Financial insecurity**	**16. I have experienced financial hardship that has affected how I feel about myself**	**0.273**	**0.325**	**0.385**	**0.806**	**0.224**
	**17. My job security has been affected by my illness**	**0.373**	**0.635**	**0.129**	**0.678**	**0.152**
	**18. I have experienced financial hardship that has affected my relationships with others**	**0.348**	**0.398**	**0.050**	**0.884**	**0.247**
**Discretionary disclosure**	**19. I do not feel I can be open with others about my illness**	**0.162**	**0.086**	**0.319**	**0.233**	**0.926**
	**20. I fear someone telling others about my illness without my permission**	**0.073**	**0.244**	**0.257**	**0.263**	**0.921**

## Discussion

The final Persian version of HIV/AIDS-related stigmascale consists of 20 items and measures internal and external HIV/AIDS stigma in five subscales: social rejection (9 items), negative self-worth (4 items), perceived interpersonal insecurity (2 items), financial insecurity (3 items), and discretionary disclosure (2 items). In this scale social rejection and financial insecurity subscales represented external stigma; negative self-worth, perceived interpersonal insecurity, and discretionary disclosure represented the internal stigma. Participants can rate their extended agreement with each item by a 5-point Likert type scale. Points were assigned as follows: 1 = strongly disagree, 2 = disagree, 3 = neutral, 4 = agree, 5 = strongly agree. Total possible scores ranged from 20 to 100. The higher score showed a stronger sense of feeling stigmatized.

Stigma is the major obstacle to an effective response to the HIV/AIDS epidemic. The complexity of HIV/AIDS-related stigma is often known as the main reason for the limited response to the phenomenon (1). To assess and describe subjective concepts such as stigma, the existence of valid tools is important (21). In addition, to assess the quality of the translation and validity process, providing comprehensive information is important. These details can help compare the results of different studies; however, most validation studies focus on psychometric tests to the translation process (22).

Hence, using both qualitative (process of translation) and quantitative methods (psychometric testing) strengthens the power of findings. In this study we used a multi-method approach in translating the HIV/AIDS related stigma scale and that is one of the main strengths of our study. This approach can decrease sources of bias in the process of translation and adaptation of the questionnaire (23).

Based on translation validity assessment, we translated the original questioner into Persian by two bilinguals. It is important that at least two qualified translators translate independently. This can provide conditions leading to the best translation (24).

Our research team conducted a focus group discussion to examine in detail the Persian version of the questionnaire to ensure the correct meaning of words such as stigma and discrimination and that such words are clearly stated in order to avoid misunderstandings. Our team consisted of two mental health counselors and one general practitioner from the Qom Consulting Clinics of Behavioral Disorders who spend a lot of time with HIV/AIDS patients and so we carried out focus group discussions with these experts in order to obtain valuable and relevant information. It was evident that conducting focus groups with experts helped to remove some words or sentences that were not clear or understandable in the viewpoint of the target group (24, 25).

After back-translation, we carried out discussions with both of the back-translators to reach a version similar to the original one. Also, psychometric properties of our study confirmed usefulness of the translated questionnaire.

To assess the internal reliability and construct validity of the HIV/AIDS-related stigma we analyzed data from 80 PLWH. Cronbach’s alpha coefficient for the overall scale was 0.85 and that showed a good internal consistency of our translated version. Also, for all the subscales Cronbach’s alpha coefficients were greater than or equal to 0.7, except for the "perceived interpersonal insecurity" subscale which was 0.57. In Li et al.'s previous studies that used this scale, similar reliability was reached. They reported Cronbach’s alpha coefficient at 0.4 for the "perceived interpersonal insecurity" subscale. This might be due to the fact that the scale contained only two items of this subscale (26).

Our analyses showed the correlation between items and their hypothesized subscale was greater than 0.5. Correlation between an item and its own subscale was significantly higher than its correlation with other subscales. Also, correlation between an item and scale other than its hypothesized scale was lower than the correlation between that item and its hypothesized scale. Hence, all items passed the test for convergent and discriminant validity.

The stigma scale with 20-items was used in this study and had good size and so could determine internal and external stigma in the five components. This provided comprehensive information on rejection, negative self-worth, perceived interpersonal insecurity, financial insecurity, and discretionary experienced in patients living with HIV/AIDS.

Whereas the stigma tool used by Ebrahimi Kalan et al., was short and in the review of stigma did not have sufficient integrity. Ebrahimi Kalan et al.'sinstrument measured three subscales in HIV-related stigma, but it does not assess internal and external stigma. They suggested future research in Iran that may explore longer versions of the stigma scale that may have meaningful subscales (18). Another questionnaire used by SeyedAlinaghi et al., was longer. Ten key areas and 45 questions made it difficult to use in a population. SeyedAlinaghi et al., indicated that applying an extensive questionnaire that required considerable amount of time to fill out as one of their study's limitations (17).

In this study a major limitation was the co-layering of stigma (related to social position, risky behavior, and so forth) which was not addressed in our scale.

The findings of this study show that the Persian version of the HIV/AIDS-related stigma scale is valid and reliable to assess HIV/AIDS-related stigma perceived by people living with HIV/AIDS in Iran.
